# The Global Burden of Disease Study Estimates of Brazil’s Cervical Cancer Burden

**DOI:** 10.5334/aogh.2756

**Published:** 2020-06-09

**Authors:** Nathalia V. S. Reis, Brenda B. Andrade, Maximiliano R. Guerra, Maria Tereza B. Teixeira, Deborah C. Malta, Valéria M. A. Passos

**Affiliations:** 1 Faculdade Ciências Médicas de Minas Gerais, BR; 2 Universidade Federal de Juiz de Fora, BR; 3 Universidade Federal de Minas Gerais, BR

## Abstract

**Background:**

Cervical cancer represents an important preventable cause of morbidity and mortality in developing countries such as Brazil. Investigating temporal evolution of a disease burden in the different realities of the country is essential for improving public policies.

**Objective:**

To describe the national and subnational burden of cervical cancer, based on the estimates of the 2017 Global Burden of Disease study.

**Methods:**

Descriptive study of premature mortality (years of life lost [YLL]) and burden of disease (disability-adjusted life years [DALYs]) associated with cervical cancer among Brazilian women aged 25–64 years, between 2000 and 2017.

**Findings:**

During the study period, age-standardized incidence decreased from 23.53 (22.79–24.26) to 18.39 (17.63–19.17) per 100,000 women, while mortality rates decreased from 11.3 (11.05–11.56) to 7.74 (7.49–8.02) per 100,000 women. These rates were about two to three times greater than equivalent rates in a developed country, such as England: 11.98 (11.45–12.55) to 10.37 (9.85–10.9), and 3.75 (3.68–3.84) to 2.82 (2.75–2.9) per 100,000 women, respectively. Poorer regions of Brazil had greater rates of the disease; for instance, Amapá State in the Northern Region had rates twice as high as the national rates during the same period. Cervical cancer was the leading cause of premature cancer-related mortality (YLL = 100.69, 91.48–110.61 per 100,000 women) among young women (25–29 years) in Brazil and eight federation units of all country regions except the Southeast in 2017. There was a decrease in the burden of cervical cancer in Brazil from 339.59 (330.82–348.83) DALYs per 100,000 women in 2000 to 238.99 (230.45–247.99) DALYs per 100,000 women in 2017.

**Conclusion:**

Although there has been a reduction in the burden of cervical cancer in Brazil, the rates remain high, mainly among young women. The persistence of inequalities between regions of Brazil suggests the importance of socioeconomic determinants in the burden for this cancer.

## Introduction

Although cervical cancer is considered a preventable disease, it remains a major cause of morbidity and mortality worldwide, especially in developing countries. In 2018, 569,847 new cases of cervical cancer were estimated globally, with an age-standardized incidence rate of 13.1/100,000 women [1]. Between 1990 and 2015, cervical cancer was a leading cause of death among women, despite a marked decrease of mortality rates by the disease over the period [2].

In Brazil in 2018, 16,370 new cases of cervical cancer were reported, with a crude incidence of 17.11 cases per 100,000 women, which made it the third most frequent tumor in the Brazilian female population. In 2016, the national mortality rate by cervical cancer was 4.70/100,000 women, which was lower compared to the global rate of 6.9/100,000 women [1,3,4]. However, there are important inequalities between Brazilian regions, which may account for the overall low rate. In the North, cervical cancer was the leading cause of cancer death, with the mortality rate of 11.07/100,000 women, roughly double that of cervical cancer-specific global mortality rate in 2016 [3,4]. These inequalities are longstanding, with temporal analysis revealing high mortality rates by this cancer, in the less developed regions of Brazil, northern and northeastern, since 1980 [5,6].

Studies of disease epidemiology have traditionally quantified incidence and mortality. However, in recent decades the survival rate of cancer patients has significantly increased with advances in the prevention, diagnosis, and treatment of this disease [7]. It is essential to investigate the disease burden of women who survive cancer, especially when considering a kind of cancer that affects women, including the ones in reproductive age, such as cervical cancer, increasing the risk of premature mortality and early morbidity.

To the best of the authors’ knowledge, to-date, there have been no studies evaluating the burden for cervical cancer in Brazil. Previous studies involving small samples, which may not be representative of the general population, assessed the impact of disease and treatment on quality of life and specific outcomes, such as sexual dysfunction [8,9].

The Global Burden of Disease (GBD) study, launched in 1990, proposed a new paradigm for health loss assessment that went beyond the numerical description of events. Years lost due to premature death are expressed with the YLL metric, results from the multiplication of each year of life lost by each cervical cancer death by the maximum life expectancy. The impact of morbidity is represented by years of disability or YLD, resulting from the prevalence of each sequela produced by a disease, multiplied by the weight of disability for that sequel. The metric representing the global burden of disease is DALY, disability-adjusted years of life lost to premature death, which is a sum of YLL and YLD. In addition, the GBD study has a standardized methodology for estimating disease burden, which allows comparability of disease burden across sites and over time [10,11,12].

Investigating temporal evolution of a disease burden in the different realities of the country is essential for improving public policies and directing resources to improve access to health, either for screening or treatment. This study aimed to describe the burden of cervical cancer in Brazil at a national and subnational level, during 2000–2017, based on estimates from the GBD study.

## Materials and Methods

This is a descriptive study of the burden of cervical cancer in the Brazilian female population, aged 25 to 64 years, conducted between 2000 and 2017. This study describes the GBD-2017 estimates of the burden of cervical cancer obtained thanks to the collaboration of a Brazilian network of researchers, the Brazil Ministry of Health, and the Institute of Health Metrics and Evaluation (IHME) of the University of Washington. Since 2015, the GBD-Brazil network of collaborators provides support and evaluate the estimates of the GBD study at the subnational level [13]. The study’s methodology has been previously described [10,11,12]. All estimates, as well as the figures, were obtained from the Global Burden of Disease 2017 (GBD 2017), available at http://ghdx.healthdata.org/gbd-results-tool.

Cervical cancer was defined according to the International Classification of Diseases 10^th^ edition (ICD-10) (codes: C53.0, C53.1, C53.8, C53.9, D06.0, D06.1, D06.7, D06.9, D26.0) [12].

All Brazilian data sources are available at: http://ghdx.healthdata.org/gbd-2017/data-input. The Brazilian Institute of Geography and Statistics (IBGE) provides the population estimates. The Mortality Information System (SIM) is responsible for the collection, storage, and evaluation process of death registries in the country, and was the main source of data. Mortality estimates were available after correcting for underreporting and for garbage codes. The detailed steps of modeling are available and already published [12].

Incidence data were obtained from the National Cancer Institute (INCA) and the Cancer Incidence in Five Continents (CI5) from the International Agency Association of Cancer Registries. The Disease Modulation software, version 2.1 (DisMod-MR 2.1) provides standardized modeling of prevalence, incidence, and YLD to each sex, age group, location, and year by using Bayesian models of meta-regression with covariates to adjust the estimates [14].

In the present study, estimates of incidence, mortality, YLL, YLD, and DALY were analyzed. The absolute values for each metric were presented with corresponding 95% uncertainty intervals (UI). UIs differ from confidence intervals, which reflect the sampling error, as they include the uncertainties of all modeling sources and steps. To determine the UIs, all GBD study metric calculations were made 1000 times, and the 95% uncertainty limits for each variable of interest were defined by the 25th and 97.5th values of the 1000 estimates [10,11,12].

For magnitude evaluation, the burden of cervical cancer in Brazil was compared to that of other five countries and 10 states of the federation, classified according to the sociodemographic index (SDI) in 2017. SDI is a composite development indicator strongly related to health outcomes, which aggregates three variables: fertility rate before the age of 25 years, per capita income, and average education attainment of the population over 15 years old. The scores range from 0 (lower income, lower education, and higher fertility) to 1 (higher income, higher education, and lower fertility). According to the SDI value, locations are classified as high, medium-high, medium, medium-low, and low development level [15].

From 2000 to 2017, all countries and Brazilian States maintained the same classification of SDI (data not shown). We included two high SDI countries [Canada (0.8820) and England (0.8488)], two medium-high SDI countries [Argentina (0.7101) and Uruguay (0.7067)], and a medium SDI country (Cuba, SDI = 0.6876). Canada, England, and Cuba were selected because they have similar public health systems as Brazil, while Uruguay and Argentina were selected for their geographical and cultural proximity.

**Figure 1:**
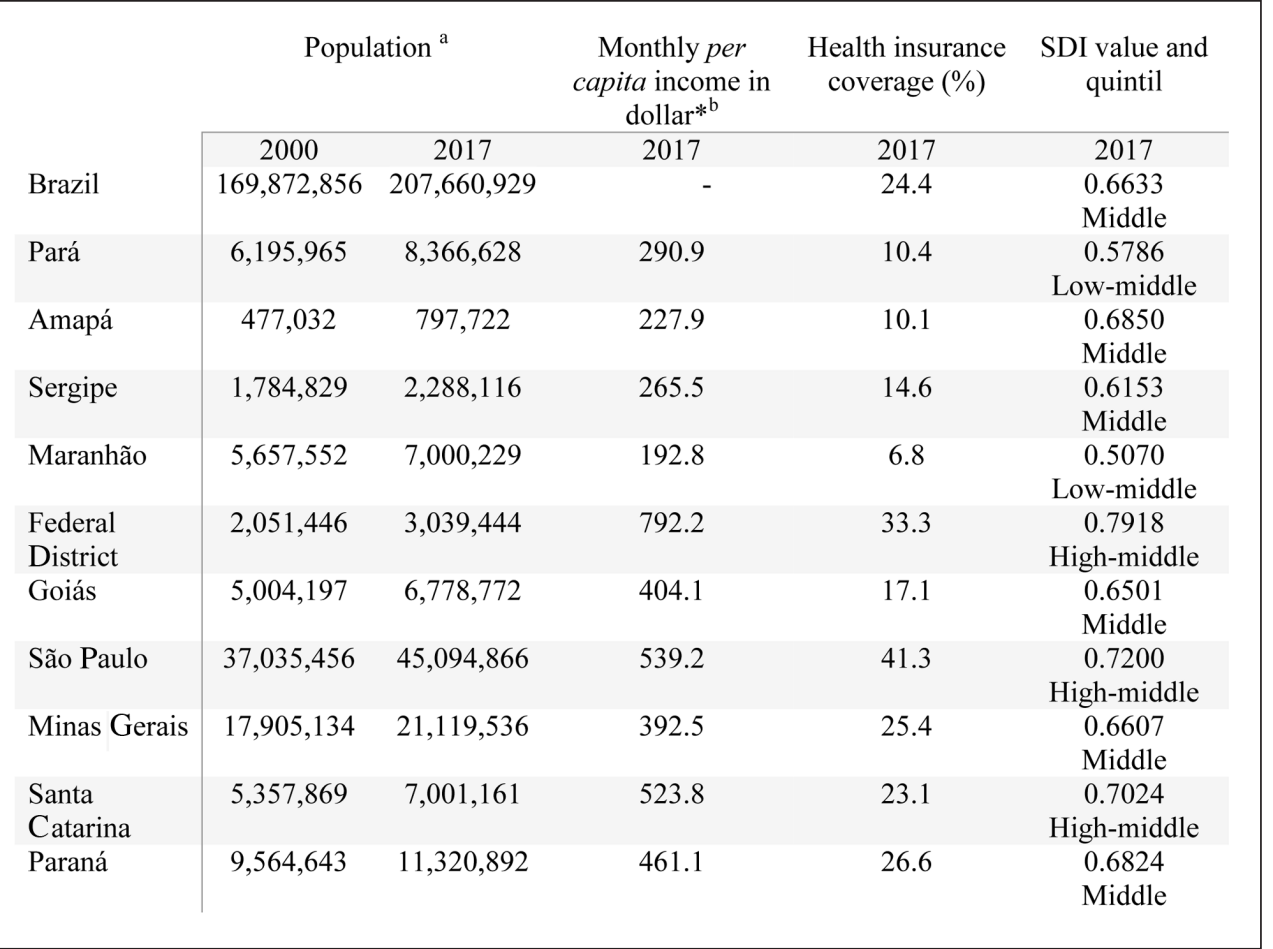
Sociodemographic characteristics of Brazil and States, in 2000 and 2017. *(1USD$ = 3.19 reais). a IBGE- available at: https://www.ibge.gov.br/estatisticas/sociais/populacao. b Institute of Applied Economic Research- available at: http://www.ipeadata.gov.br. c National Health Agency- available at: http://www.ans.gov.br/anstabnet/cgi-bin/tabnet?dados/tabnet_tx.def, dez.2017.

To allow comparison among the five Brazilian regions, we analyzed the estimates of two states within each region, defined as the highest and lowest SDI in each geographic region, in at least two of the considered years (2000, and 2010 or 2017): North (Amapá and Pará), Northeast (Sergipe and Maranhão), Midwest (Federal District and Goiás), Southeast (São Paulo and Minas Gerais), and South (Santa Catarina and Paraná) (Figure 1).

## Results

In Brazil, there was a decrease (–1.45%) in the age-standardized estimated incidence rates from 23.53 (95% UI 22.79–24.26) per 100,000 women in 2000 to 18.39 (95% UI 17.63–19.17) per 100,000 women in 2017 (Table 1).

**Table 1:** Estimated age-standardized incidence and mortality rates per 100,000 women for cervical cancer in 2000 and 2017.

Country	2000	2017	Δ 2000 A 2017^a^

	Incidence		

Brazil	23.53 (22.79–24.26)	18.39 (17.63–19.17)	–1.45%
Cuba	25.20 (22.36–28.31)	19.4 (15.91–23.36)	–1.54%
Argentina	32.94 (29.24–36.87)	31.67 (25.76–38.25)	–0.23%
Uruguay	33.11 (29.57–37.34)	29.15 (23.4–35.61)	–0.75%
Canada	10.3 (9.17–11.58)	9.37 (7.88–10.94)	–0.55%
England	11.98 (11.45–12.53)	10.37 (9.85–10.9)	–0.85%
	**Mortality**		

Brazil	11.30 (11.05–11.56)	7.74 (7.49–8.02)	–2.22
Cuba	9.10 (8.69–9.54)	7.32 (6.19–8.59)	–1.28
Argentina	12.38 (11.76–12.97)	10.82 (9.09–12.57)	–0.80
Uruguay	13.45 (12.72–14.19)	10.70 (8.97–12.45)	–1.35
Canada	3.58 (3.47–3.70)	2.86 (2.58–3.16)	–1.33
England	3.75 (3.68–3.84)	2.82 (2.75–2.9)	–1.68

^a^ Annual % change. IHME, GBD 2017. Available at: http://ghdx.healthdata.org/gbd-results-tool.

The number of estimated age-standardized mortality rate decreased from 11.3 (95% UI 11.05–11.56) per 100,000 women in 2000 to 7.74 (95% UI 7.49–8.02) per 100,000 women in 2017.

Compared to other countries, Brazil had cervical cancer incidence rate that was double the rate reported for the high SDI countries, smaller than the medium-high SDI countries, and similar to the medium SDI country. Nevertheless, throughout the period, the mortality rate was three times higher in Brazil compared to Canada and England. Concurrently, while Cuba showed similar mortality rates, Argentina and Uruguay had higher mortality rates compared to Brazil (Table 1).

In the subnational analysis, the states of the South and Southeast regions had lower rates of incidence and mortality for cervical cancer, which were likely to skew national rates due to population density. Incidence and mortality rates in the states of the North and Northeast were consistently higher, reaching double or triple the national rate (Table 2). For example, the national incidence rate was 18.39 (95% UI 17.63–19.17) per 100,000 in 2017, while in the State of Amapá was 37.47 (95% UI 32.94–42.40) in the same year (Table 2).

**Table 2:** Estimated incidence and mortality rate per 100,000 women for cervical cancer in Brazil and Federative Units by age group in 2000 and 2017.

LOCALITY	Age groups (years)	Incidence			Mortality		

		2000	2017	Δ 2000–2017^a^	2000	2017	Δ 2000–2017^a^

BRAZIL	ASR	23.53 (22.79–24.26)	18.39 (17.63–19.17)	–1.45%	11.3 (11.05–11.56)	7.74 (7.49–8.02)	–2.22%
	25–29	13.89 (12.22–15.63)	15. 38 (13.46–17.81)	0.60%	1.71 (1.58–1.85)	1.66 (1.51–1.83)	–0.18%
	30–34	29.14 (26.02–32.33)	30.91 (27.56–34.75)	0.35%	4.30 (4.03–4.58)	4.06 (3.77–4.38)	–0.33%
	35–39	39.43 (35.38–43.97)	35.92 (32.21–40.01)	–0.55%	8.24 (7.77–8.76)	6.80 (6.28–7.28)	–1.13%
	40–44	47.61 (42.94–52.73)	37.14 (33.66–41.66)	–1.46%	14.16 (13.45–14.89)	10.02 (9.41–10.68)	–2.03%
	45–49	49.31 (45.50–54.06)	36.58 (33.42–40.26)	–1.76%	19.29 (18.42–20.26)	13.09 (12.38–13.89)	–2.28%
	50–54	51.39 (47.40–55.69)	35.50 (32.61–38.65)	–2.18%	25.01 (23.75–26.30)	16.19 (15.29–17.24)	–2.56%
	55–59	58.26 (54.20–62.85)	38.46 (35.52–41.88)	–2.44%	30.64 (29.19–32.28)	19.23 (17.97–20.55)	–2.74%
	60–64	57.89 (53.95–62.33)	36.77 (33.79–40.01)	–2.67%	35.23 (33.43–37.14)	21.49 (20.12–23.07)	–2.91%
NORTH							

AMAPÁ	ASR	46.57 (41.36–52.08)	37.47 (32.94–42.40)	–1.28%	22.47 (20.81–24.12)	16.62 (15.18–18.08)	–1.77%
	25–29	34.59 (20.56– 53.00)	24.45 (14.20 – 38.54)	–2.04%	4.34 (2.97–6.00)	2.76 (1.83–3.96)	–2.65%
	30–34	74.87 (45.39–118.50)	64.18 (39.34–96.24)	–0.91%	11.17 (8.15 –14.49)	8.58 (6.12–11.16)	–1.55%
	35–39	72.25 (46.80–105.35)	68.02 (44.63–101.62)	–0.35%	15.50 (11.82–19.70)	13.25 (9.99–16.62)	–0.92%
	40–44	91.65 (63.54–127.38)	72.48 (49.42–103.65)	–1.38%	28.04 (22.22–34.36)	20.19 (15.70–24.99)	–1.93%
	45–49	100.46 (72.59–137.58)	72.61 (50.65–100.74)	–1.91%	40.22 (31.88–48.96)	26.68 (20.72–33.91)	–2.41%
	50–54	88.50 (66.06–118.95)	68.81 (49.73–93.47)	–1.48%	43.75 (35.25–52.64)	31.95 (24.41–39.49)	–1.85%
	55–59	105.93 (78.79–140.00)	89.25 (63.85–118.55)	–1.01%	56.74 (44.80–70.34)	46.15 (35.67–58.18)	–1.22%
	60–64	102.60 (74.38–132.56)	85.91 (64.91–114.61)	–1.04%	63.74 (49.07–80.14)	51.24 (40.16–64.74)	–1.28%
PARÁ	ASR	38.43 (34.39–43.00)	29.05 (26.00–33.04)	–1.65%	17.93 (16.76–19.14)	12.70 (11.74–13.83)	–2.03%
	25–29	26.55 (16.57–40.79)	24.85 (14.91–39.19)	–0.39%	3.37 (2.50–4.27)	2.85 (2.03–3.93)	–0.98%
	30–34	48.97 (32.14–72.03)	44.93 (29.31–67.46)	–0.51%	7.58 (6.03–9.28)	6.31 (4.80–8.15)	–1.08%
	35–39	71.51 (49.49–102.61)	55.14 (35.76–80.61)	–1.53%	15.62 (12.70–18.82)	11.01 (8.65–13.61)	–2.06%
	40–44	81.19 (57.73–109.74)	58.14 (40.22–82.59)	–1.96%	25.27 (21.13–29.57)	16.55 (13.16–20.39)	–2.49%
	45–49	85.52 (64.99–111.43)	60.93 (44.00–82.64)	–1.99%	34.88 (29.85–40.74)	22.94 (18.39–27.61)	–2.47%
	50–54	81.53 (63.44–103.84)	55.72 (41.26–73.70)	–2.24%	41.29 (34.46–47.94)	26.63 (21.25–32.07)	–2.58%
	55–59	90.44 (71.17–116.68)	63.76 (47.63–84.79)	–2.06%	49.10 (41.29–58.40)	33.25 (26.51–40.42)	–2.29%
	60–64	87.77 (69.42–112.23)	62.44 (47.41–80.72)	–2.00%	54.79 (46.47–64.41)	37.77 (30.17–46.77)	–2.19%
NORTHEAST							

MARANHÃO	ASR	38.83 (33.64–45.33)	29.12 (25.35–33.17)	–1.69%	17.15 (15.36–19.68)	12.29 (11.26–13.44)	–1.96%
	25–29	29.16 (16.87–46.59)	22.84 (12.92–36.02)	–1.44%	3.79 (2.51–5.51)	2.68 (1.82–3.71)	–2.03%
	30–34	55.65 (35.05–84.19)	47.49 (30.43–72.06)	–0.93%	8.71 (6.28–11.79)	6.84 (5.06–9.00)	–1.42%
	35–39	82.15 (52.47–122.35)	58.74 (38.28–85.25)	–1.97%	18.38 (13.68–23.73)	12.09 (9.34–15.39)	–2.46%
	40–44	84.82 (57.67–124.79)	60.32 (41.81–83.28)	–2.01%	27.04 (20.95–34.45)	17.68 (13.97–21.56)	–2.50%
	45–49	92.97 (67.12–125.42)	69.09 (49.98–92.84)	–1.75%	38.44 (30.31–48.80)	26.63 (21.14–32.37)	–2.16%
	50–54	81.08 (59.98–109.79)	65.01 (48.72–86.35)	–1.30%	41.70 (33.01–52.78)	31.67 (26.03–38.24)	–1.62%
	55–59	88.08 (63.37–119.53)	60.49 (45.13–79.14)	–2.21%	48.48 (37.49–61.32)	31.90 (25.43–39.01)	–2.46%
	60–64	86.08 (64.20–115.40)	53.92 (40.42–71.31)	–2.75%	54.65 (42.51–69.37)	33.31 (26.37–41.64)	–2.91%
SERGIPE	ASR	29.38 (26.42–32.57)	22.11 (19.46–25.25)	–1.67%	13.85 (12.96–14.70)	9.83 (9.00–10.80)	–2.02%
	25–29	17.18 (10.55–26.24)	14.36 (8.05–23.57)	–1.05%	2.25 (1.62–3.06)	1.63 (1.04–2.36)	–1.87%
	30–34	41.32 (26.48–60.82)	35.27 (21.61–54.25)	–0.93%	6.44 (4.79–8.38)	4.85 (3.48–6.43)	–1.67%
	35–39	52.09 (34.83–75.60)	39.79 (25.58–62.73)	–1.58%	11.60 (9.21–14.46)	7.87 (5.86–10.36)	–2.28%
	40–44	66.49 (48.45–89.31)	49.53 (33.69–70.81)	–1.73%	20.84 (17.32–25.12)	13.90 (10.65–17.52)	–2.38%
	45–49	60.84 (44.38–81.91)	43.81 (32.21–60.34)	–1.93%	24.61 (20.03–29.52)	16.27 (12.92–20.29)	–2.44%
	50–54	68.83 (53.37–89.75)	45.32 (32.98–62.52)	–2.46%	34.82 (28.86–41.28)	21.26 (16.82–26.54)	–2.90%
	55–59	69.18 (53.16–89.92)	50.70 (37.73–67.17)	–1.83%	37.63 (31.20–45.35)	26.20 (20.94–32.47)	–2.13%
	60–64	65.41 (50.44–82.91)	41.48 (31.02–55.88)	–2.68%	40.90 (33.55–49.31)	24.97 (19.44–31.99)	–2.90%
MIDWEST							

DISTRITO FEDERAL	ASR	25.85 (23.38–28.58)	16.23 (14.36–18.42)	–2.74%	13.02 (12.29–13.86)	6.87 (6.28–7.54)	–3.76%
	25–29	13.75 (8.33 –22.17)	11.96 (6.77–19.51)	–0.82%	1.53 (1.08–2.10)	1.09 (0.72–1.56)	–2.01%
	30–34	35.31 (22.07–53.46)	26.87 (15.98–41.77)	–1.61%	4.68 (3.51–6.08)	3.01 (2.08–4.12)	–2.59%
	35–39	32.33 (21.64–46.79)	29.53 (18.17–45.14)	–0.53%	6.24 (4.89–7.81)	4.80 (3.55–6.31)	–1.53%
	40–44	43.42 (30.78–60.17)	31.54 (21.22–45.35)	–1.88%	11.91 (9.78–14.23)	7.40 (5.76–9.56)	–2.80%
	45–49	46.60 (34.22–62.71)	33.08 (22.83–46.63)	–2.01%	17.06 (14.15–20.20)	10.43 (8.11–13.10)	–2.89%
	50–54	49.33 (37.14–65.05)	30.08 (21.20–41.36)	–2.91%	22.73 (18.56–27.43)	12.34 (9.69–15.31)	–3.59%
	55–59	72.09 (54.15–94.32)	32.20 (23.66–44.16)	–4.74%	35.93 (29.46–42.54)	14.64 (11.43–18.20)	–5.28%
	60–64	73.92 (55.88–97.68)	30.17 (22.06–41.45)	–5.27%	40.90 (33.55–49.31)	24.97 (19.44–31.99)	–2.90%
GOIÁS	ASR	25.37 (23.04–27.83)	17.59 (15.50–20.03)	–2.15%	12.62 (11.96–13.25)	7.48 (6.88–8.12)	–3.08%
	25–29	13.54 (7.97–20.66)	13.10 (7.67–21.02)	–0.19%	1.66 (1.18– 2.19)	1.42 (0.97–2.03)	–0.91%
	30–34	30.52 (20.00–44.97)	30.98 (19.58–46.48)	0.089%	4.45 (3.49–5.49)	4.12 (2.98–5.45)	–0.46%
	35–39	36.43 (25.25–51.74)	34.34 (22.82–50.53)	–0.35%	7.64 (6.20–9.29)	6.51 (5.02–8.31)	–0.94%
	40–44	51.25 (36.91–70.90)	34.35 (23.48–48.34)	–2.35%	15.31 (12.75–18.06)	9.34 (7.36–11.68)	–2.91%
	45–49	56.63 (42.41–74.28)	35.92 (25.86–49.02)	–2.68%	22.12 (18.69–25.72)	12.90 (10.19–16.00)	–3.17%
	50–54	55.14 (42.66–72.08)	33.44 (24.54–44.65)	–2.94%	26.79 (22.84–31.18)	15.27 (12.09–18.70)	–3.31%
	55–59	61.97 (47.80–79.40)	36.61 (26.41–49.12)	–3.10%	32.46 (27.37–38.01)	18.44 (14.52–22.68)	–3.33%
	60–64	63.45 (49.72–80.54)	36.33 (27.10–49.53)	–3.28%	38.62 (32.04–46.09)	21.38 (17.11–26.68)	–3.48%
SOUTHEAST							

SÃO PAULO	ASR	19.93 (18.37–21.67)	13.79 (12.20–15.56)	–2.17%	10.14 (9.71–10.59)	5.79 (5.39–6.23)	–3.30%
	25–29	9.96 (6.39–15.18)	13.40 (7.98–21.77)	1.74%	1.15 (0.88–1.46)	1.33 (0.97–1.77)	0.83%
	30–34	20.23 (13.15–30.01)	22.39 (13.95–34.18)	0.60%	2.82 (2.27–3.45)	2.73 (2.05–3.50)	–0.18%
	35–39	28.37 (19.64–39.77)	25.43 (16.61–37.01)	–0.64%	5.60 (4.66–6.64)	4.50 (3.46–5.73)	–1.29%
	40–44	36.73 (26.39–49.78)	25.14 (17.23–36.38)	–2.23%	10.38 (8.93–12.07)	6.34 (5.00–7.95)	–2.90%
	45–49	38.71 (28.96–51.24)	24.09 (17.13–34.21)	–2.79%	14.56 (12.43–16.88)	8.09 (6.51–9.87)	–3.45%
	50–54	44.09 (34.80–56.20)	25.76 (19.09–34.93)	–3.16%	20.64 (17.87–23.80)	11.18 (9.07–13.76)	–3.61%
	55–59	52.51 (41.37–67.51)	29.86 (21.85–40.27)	–3.32%	26.77 (23.08–31.11)	14.35 (11.53–17.99)	–3.67%
	60–64	53.92 (42.76–67.29)	29.11 (21.82–38.79)	–3.63%	31.93 (27.18–37.46)	16.33 (12.97–20.54)	–3.94%
MINAS GERAIS	ASR	17.90 (16.26–19.62)	14.18 (12.63–16.03)	–1.37%	8.98 (8.50–9.46)	6.26 (5.82–6.71)	–2.12%
	25–29	9.29 (6.04–14.17)	10.46 (6.23–16.12)	0.70%	1.15 (0.86–1.49)	1.13 (0.78– 1.56)	–0.10%
	30–34	19.88 (12.85–29.20)	22.42 (13.89–34.79)	0.71%	2.93 (2.28–3.63)	2.88 (2.14–3.81)	–0.086%
	35–39	28.15 (19.12–39.89)	26.20 (17.03–40.20)	–0.42%	5.83 (4.69–7.19)	4.85 (3.71–6.20)	–1.08%
	40–44	34.12 (24.64–47.20)	25.27 (17.17–36.67)	–1.77%	10.15 (8.35–12.37)	6.71 (5.30–8.62)	–2.43%
	45–49	36.59 (27.33–48.23)	28.07 (19.74–40.04)	–1.56%	14.21 (11.93–16.83)	9.80 (7.79–12.43)	–2.19%
	50–54	39.80 (30.56–51.99)	28.81 (21.10–39.31)	–1.90%	19.42 (16.24–23.23)	12.98 (10.51–15.84)	–2.37%
	55–59	55.89 (43.34–71.87)	36.22 (26.48–48.45)	–2.55%	23.37 (19.50–28.28)	15.40 (12.25–19.11)	–2.46%
	60–64	46.28 (36.85–57.93)	31.40 (23.40–40.64)	–2.28%	28.11 (23.64–33.54)	18.21 (14.35–22.72)	–2.55%
SOUTH							

PARANÁ	ASR	21.90 (19.99–23.92)	17.54 (15.40–19.87)	–1.31%	10.67 (10.13–11.24)	7.34 (6.83–7.96)	–2.20%
	25–29	12.92 (8.06–19.69)	13.86 (8.37–22.21)	0.42%	1.56 (1.17–2.07)	1.46 (1.01–2.09)	–0.39%
	30–34	27.66 (18.22–40.49)	30.57 (19.19–47.24)	0.59%	4.04 (3.21–5.07)	3.90 (2.95–4.99)	–0.22%
	35–39	37.51 (25.79–54.26)	35.71 (23.27–53.24)	–0.29%	7.77 (6.34–9.38)	6.55 (5.02–8.53)	–1.00%
	40–44	40.45 (29.17–57.51)	35.69 (24.75–49.89)	–0.74%	12.01 (10.01–14.25)	9.42 (7.53–11.62)	–1.43%
	45–49	43.78 (32.58–57.63)	32.18 (22.66–43.94)	–1.81%	17. 12 (14.34–20.06)	11.30 (9.14–13.95)	–2.45%
	50–54	48.21 (37.48–62.77)	34.08 (25.08–46.38)	–2.04%	23.48 (19.94–27.63)	15.43 (12.53–18.75)	–2.47%
	55–59	55.89 (43.34–71.87)	36.22 (26.48–48.45)	–2.55%	29.34 (24.62–34.81)	17.92 (14.23–22.54)	–2.90%
	60–64	56.27 (44.27–71.78)	34.68 (25.70–45.88)	–2.85%	34.21 (28.52–40.79)	20.04 (16.06–24.90)	–3.15%
SANTA CATARINA	ASR	20.87 (18.92–23.21)	16.60 (14.60–18.97)	–1.35%	9.74 (9.17–10.26)	6.75 (6.22–7.33)	–2.15%
	25–29	10.36 (6.24– 16.43)	12.79 (7.26–20.94)	1.24%	1.20 (0.85–1.64)	1.28 (0.84–1.82)	0.36%
	30–34	26.08 (17.18–38.00)	28.07 (17.38–42.51)	0.43%	3.66 (2.81–4.66)	3.42 (2.45–4.53)	–0.40%
	35–39	37.32 (25.29–54.00)	34.84 (22.21–50.50)	–0.40%	7.50 (6.01–9.29)	6.13 (4.69–7.91)	–1.19%
	40–44	47.95 (33.17–66.45)	37.43 (25.34–54.50)	–1.46%	13.76 (11.38–16.73)	9.47 (7.39–11.82)	–2.20%
	45–49	46.24 (34.29–61.38)	31.86 (22.16–43.73)	–2.19%	17.43 (14.33–20.62)	10.81 (8.51–13.35)	–2.81%
	50–54	46.23 (34.59–61.43)	31.33 (22.74–42.98)	–2.29%	21.87 (17.98–26.17)	13.71 (10.75–16.65)	–2.75%
	55–59	48.41 (37.00–63.02)	32.07 (22.72–43.50)	–2.42%	24.81 (20.26–29.56)	15.44 (12.11–19.20)	–2.79%
	60–64	47.86 (37.04–63.45)	31.21 (23.11–41.87)	–2.52%	28.42 (23.20–34.37)	17.74 (14.01–22.08)	–2.77%

^a^ Annual % change ASR- Age-standardized rate. IHME, GBD 2017. Available at: http://ghdx.healthdata.org/gbd-results-tool.

The incidence of cervical cancer appeared to increase with age, peaking around the age of 50 years, beyond which point the risk of developing the disease tended to stabilize. For example, in 2017 cervical cancer incidence rate in Brazil more than doubled from 15.38 (95% UI 13.46–17.81) in the age group of 25–29 years old to 38.46 (95% UI 35.52–41.88) in the age group of 55–59 years old.

It is worth noting the small positive variation (increase) in the incidence rates nationwide in Brazil and specifically in the states of São Paulo, Minas Gerais, Paraná, and Santa Catarina in women aged 25–34 years old, which is in contrast to the pattern of negative variation (decrease) observed in the other states across age groups.

Regarding mortality rates, there has been an overall fall, except for isolated and non-significant increases in the states of São Paulo and Santa Catarina recorded in the age group of 25–29 years old in both states. With age, cervical cancer mortality increased about 12-fold from 1.66 (95% UI 1.51–1.83) in the age group of 25–29 years old to 21.49 (95% UI 20.12–23.07) in the age group of 60–64 years old in 2017 (Table 2).

**Figure 2:**
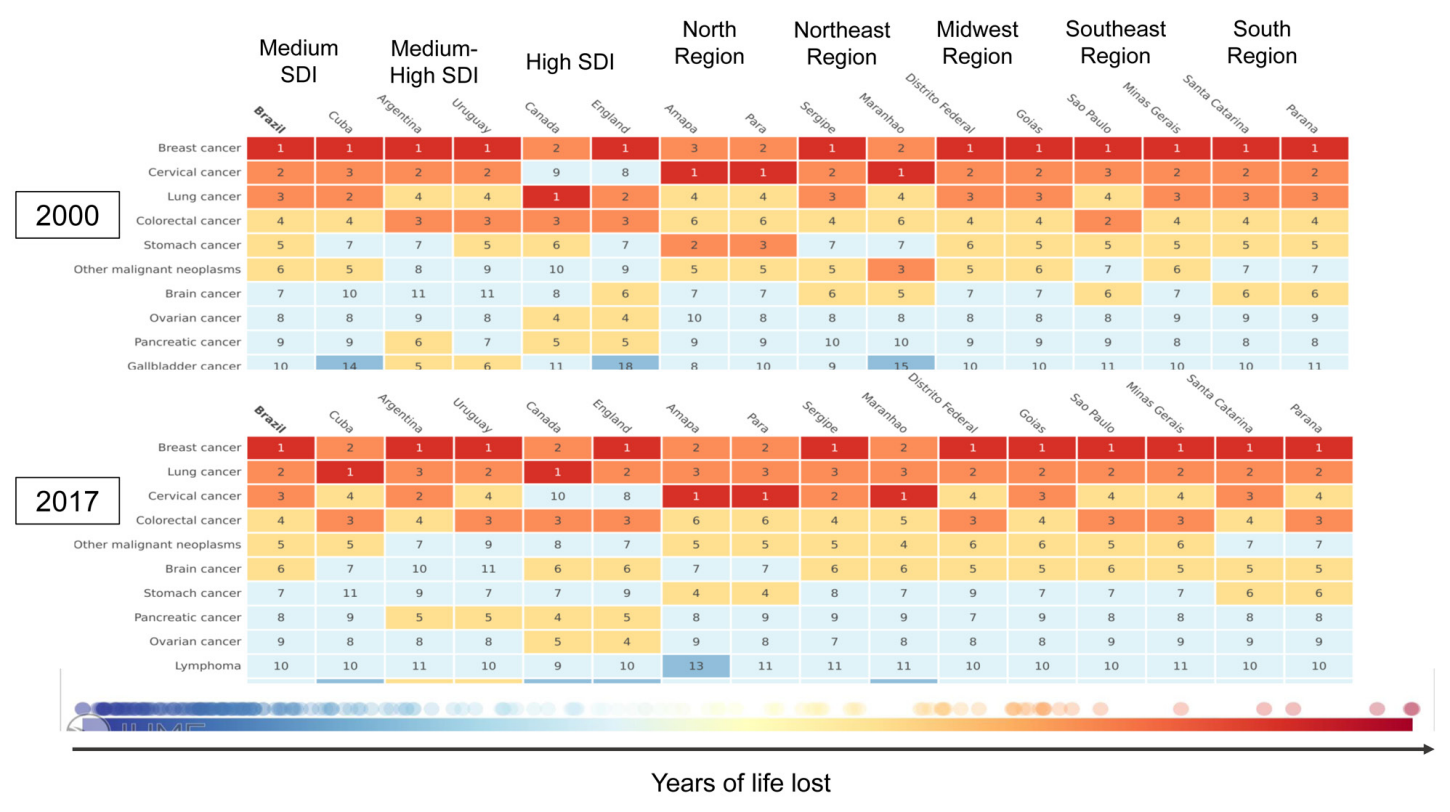
Ranking of premature deaths (age-standardized YLL) by cervical cancer, 2000 and 2017. GBD 2017, adapted figure.

Cervical cancer has moved from the second (YLL = 328.8, 95% UI 321.55–336.83) to the third leading cause (YLL = 229.8, 95% UI 222.14–238.18) of premature death due to cancer during the study period. In 2017 in England and Canada, cervical cancer appeared as the ninth and eleventh leading cause of premature death due to cancer, respectively. In 2017 in Brazil, it was the leading cause of premature cancer-related mortality in the states of Amapá, Pará, and Maranhão (Figure 2).

Among young women aged 25–29 years in 2017, cervical cancer was the leading cause of premature cancer-related mortality in Brazil (YLL = 100.69, 95% UI 91.48–110.61), in particular, in the states of Amapá (YLL = 167.18, 95% UI 110.53–239.54), Goiás (YLL = 86.19, 95% UI 58.91–122.89), Maranhão (YLL = 162.61, 95% UI 110.52–224.41), Paraná (YLL = 88.69, 95% UI 61.19–126.43), Sergipe (YLL = 98.98, 95% UI 63.13–143.11), Santa Catarina (YLL = 77.33, 95% UI 50.92–110.17), and Pará (YLL = 172.65, 95% UI 123.16–238.25).

The disability burden measured by YLD remained stable during 2000–2017 in Brazil. In 2000, cervical cancer was the second leading cause of cancer-related disability per 100,000 women (YLD = 10.79, 95% UI 7.82–14.21), and remained such in 2017 (YLD = 8.89, 95% UI 6.47–11.84). In the states of Amapá and Maranhão, as for premature mortality, cervical cancer was the leading cause of disability in 2017.

**Figure 3:**
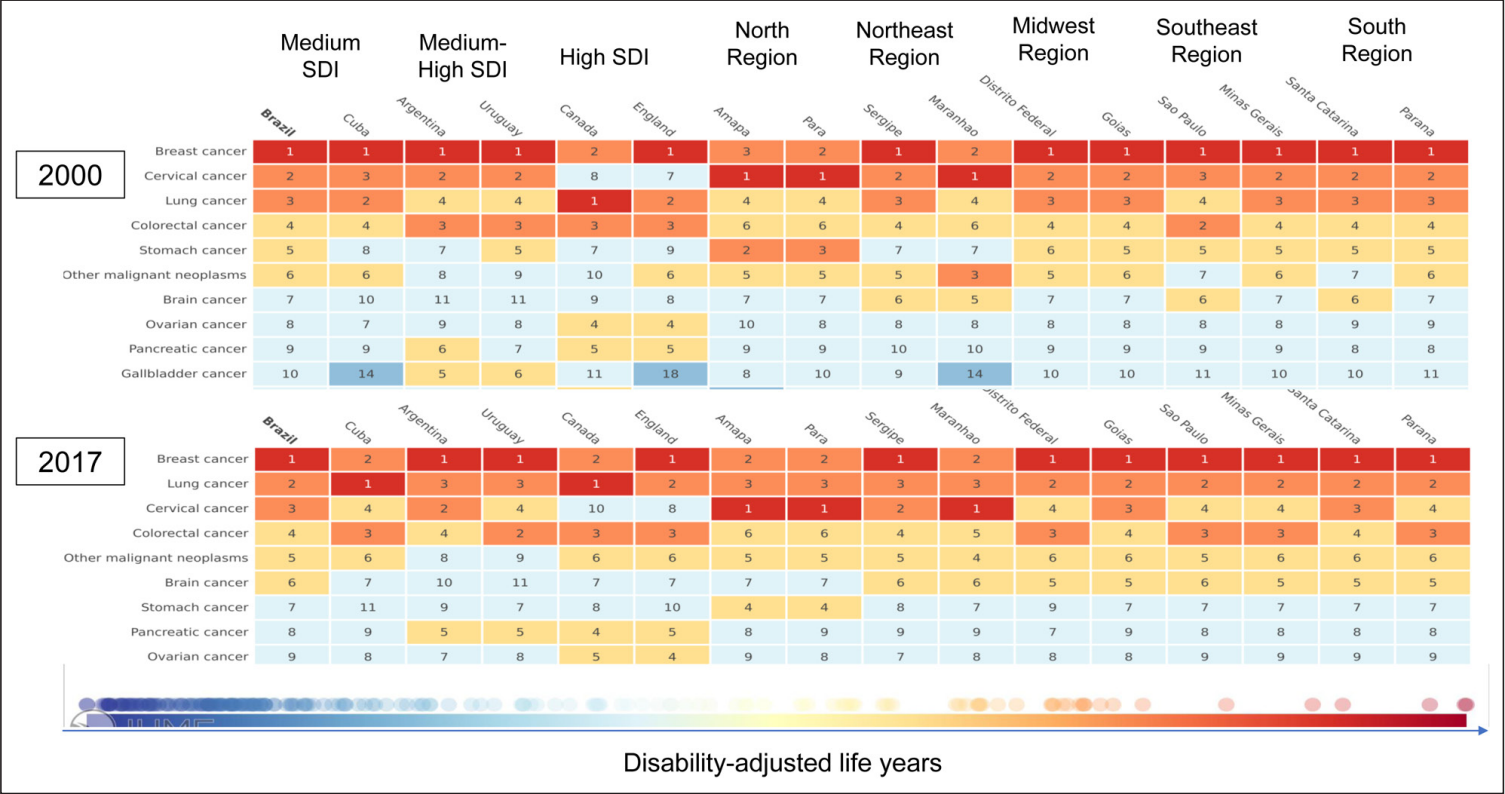
Ranking of the burden of disease (age-standardized DALYs) by cervical cancer, 2000 and 2017. GBD 2017, adapted figure.

Between 2000 and 2017, there was a reduction in the burden of cervical cancer in Brazil from 339.59 (95% UI 330.82–348.83) to 238.99 (95% UI 230.45–247.99) DALYs per 100,000 women, making cervical cancer the third leading cause of years lost due to premature disability-adjusted death. Overall, the burden of cervical cancer was high in Brazil compared to countries such as England and Canada, where, in 2017, cervical cancer appeared as the ninth and eleventh most burdensome disease. Specifically, DALYs per year per 100,000 women in these countries were 82.53 (95% UI 73.66–92.32) in Canada and 86.32 (95% UI 83.32–89.53) in England (Figure 3).

## Discussion

At the beginning of the new century, despite a general downward trend on the rates evaluated, this study shows that cervical cancer remains an important cause of morbidity and mortality among Brazilian women, including the ones in the reproductive age. Additionally, socioeconomic inequalities that persist in Brazil tends to translate into higher disease rates in poorer regions of the country, such as the Northern and Northeastern regions [2].

Brazilian estimates of incidence and mortality were about twice as high as estimates of developed countries, suggesting socioeconomic determinants may play a role in the burden of cervical cancer. Furthermore, countries with comparable SDIs, such as Brazil and Cuba, both having public health systems, had lower rates than other countries in Latin America; this suggests that access to healthcare may also be a determinant of the burden of this disease.

In 2011, the Brazilian Ministry of Health launched the ‘Strategic Action Plan to Tackle Non-Communicable Chronic Diseases (NCD) in Brazil, 2011–2022’ [16]. The expansion of primary healthcare has resulted in increased access to the Pap smear, although its coverage remains less than adequate, especially in some states of the country.

The World Health Organization (WHO) considers suitable screening coverage of 80% of the sexually active female population aged 25–64 years with a protocol-compliant pap smear [17]. In 2013, a national survey showed the self-reported coverage of pap smears in women aged 25 to 64 years was 78.8%. In addition, women with private health insurance are three times more likely to have access to pap smear than women who depend on the public health system [18]. Despite the expansion of the coverage of pap smear, in Brazil, as in other developing countries, opportunistic screening still predominates. It is estimated that the implementation of organized screening, as opposed to opportunistic one, may reduce mortality by 80% [19].

As an additional cervical cancer prevention strategy, since 2014, HPV vaccination has been available in the national vaccine program. At the time of writing, the coverage among girls is 73.6% for the first dose and 47% for the second dose, while among boys it is 25.7% for the first dose and 21.5% for the second dose [20]. Currently, it is not possible to evaluate the impact of this measure.

Although there were improvements in the prevention of cervical cancer in the country with the expansion of the screening program and the availability of the vaccine, diagnosis and access to treatment are also further determinant of the burden of cervical cancer. The higher estimates of incidence and mortality rates in the less developed regions may be related to low access to cancer health care. Late diagnosis of cervical cancer is observed in more than half Brazilian cases. Women with lower education or income, and without private health insurance are more likely to being diagnosed in an advanced stage of the disease. Furthermore, women from the most developed region (SE) have significantly less chance of late diagnosis [18,19].

Despite the high burden of disease, the overall decrease in cervical cancer incidence and mortality rates in Brazil might be linked to socioeconomic growth and increased access to preventive, diagnostic, and treatment services during the study period [21]. The Brazilian Congress recently approved a law restricting funds allocated to the health sector for the next 20 years. Restraining funds could jeopardize future improvements in health indicators [22].

It is noteworthy that there is an increase in cervical cancer incidence rates among women aged 25 to 34 years resident in more developed states such as São Paulo, Minas Gerais, Paraná, and Santa Catarina. Despite the small magnitude of the variation and the confluence of the uncertainty intervals, this phenomenon should be monitored to ascertain whether it is associated with recent reduction in prevention and treatment services for women at risk of cervical cancer.

DALYs estimates for cervical cancer in Brazil were more influenced by mortality burden (YLL) than disability burden (YLD), probably because of the small availability of morbidity data, leading to underreporting disability. The homogeneous estimates of YLD across the country may reflect the small number of studies on cervical cancer prevalence and survival rates in Brazil. It is likely that, overtime, the number of women living with cervical cancer and its consequences will increase, calling for development of policies on comprehensive and adequate care aimed at improving the quality of life for these patients.

The literature shows that the sequelae of cancer treatment range from more acute and simple problems, such as fatigue and diarrhea, to lasting problems, such as vaginal stenosis and dryness, dyspareunia, and other sexual disorders. In women with gynecological sequelae, depression, anxiety, and body image disorders related to sleep quality and stress are common [23,24,25]. In premenopausal women, infertility, and hormonal dysfunctions have been previously described [26,27].

The YLD estimate is the product of the prevalence of the disability weight of the disease. In this specific case, the GBD study used incidence sources, such as the INCA, to indirectly arrive at a prevalence estimate. Moreover, the choice of the GBD study to estimate disability weights, considering as sequelae of cervical cancer only the staging of the disease, seems insufficient as it neglects the long-term effects of treatment that might significantly impact women, for example, infertility and sexual dysfunction.

The Brazilian Mortality System has an overall 96.1% of coverage, reaching almost 100% coverage in the states of the South and Southeast regions. Nevertheless, the ill-defined causes of death are still high, ranging from 5.7% in the south to 13.5% in the North region [28]. The GBD mortality estimates presents the correction by underreporting and garbage codes as strengths, making its estimates more reliable and generally higher than that presented by databases with crude data. However, the limited availability of national data on tumor stage or sequelae restricts the analysis.

In conclusion, the high and unequal burden of cervical cancer in Brazil, together with decreasing in incidence and mortality rates, highlights the importance of implementing and maintaining investments in public health.
